# Antagonistic and Cooperative Actions of Kif7 and Sufu Define Graded Intracellular Gli Activities in Hedgehog Signaling

**DOI:** 10.1371/journal.pone.0050193

**Published:** 2012-11-16

**Authors:** Kelvin King Lo Law, Shigeru Makino, Rong Mo, Xiaoyun Zhang, Vijitha Puviindran, Chi-chung Hui

**Affiliations:** 1 Program in Developmental & Stem Cell Biology, The Hospital for Sick Children, Toronto Medical Discovery Tower, Toronto, Ontario, Canada; 2 Department of Molecular Genetics, University of Toronto, Toronto, Ontario, Canada; 3 Mutagenesis and Genomics Team, RIKEN BioResource Center, Koyadai, Tsukuba, Ibaraki, Japan; University of Queensland, Australia

## Abstract

Graded Hedgehog (Hh) signaling governs the balance of Gli transcriptional activators and repressors to specify diverse ventral cell fates in the spinal cord. It remains unclear how distinct intracellular Gli activity is generated. Here, we demonstrate that Sufu acts universally as a negative regulator of Hh signaling, whereas Kif7 inhibits Gli activity in cooperation with, and independent of, Sufu. Together, they deter naïve precursors from acquiring increasingly ventral identity. We show that Kif7 is also required to establish high intracellular Gli activity by antagonizing the Sufu-inhibition of Gli2. Strikingly, by abolishing the negative regulatory action of Sufu, diverse ventral cell fates can be specified in the absence of extracellular Hh signaling. These data suggest that Sufu is the primary regulator of graded Hh signaling and establish that the antagonistic and cooperative actions of Kif7 and Sufu are responsible for setting up distinct Gli activity in ventral cell fate specification.

## Introduction

Sonic hedgehog (Shh) acts as a classical morphogen forming a ventral-to-dorsal signaling gradient to specify diverse cell fates in the spinal cord [1]–[3]. Shh first emanates from the notochord to induce the formation of floor plate (FP) cells, which then serve as a secondary source of Shh, and patterns the ventral neural tube into five neuronal progenitor populations, p0, p1, p2, pMN and p3 [2]–[4]. Increasing signaling activity, determined by the level and duration of Shh exposure, drives naïve neuroepithelial cells to progressively more ventral neuronal cell fates. For example, Shh first induces Olig2^+^ pMN precursors, which are programmed by additional Shh signaling to become Nkx2.2^+^ p3 cells. Furthermore, a temporal requirement of Shh signaling is involved in the induction of the non-neuronal FP cells. While FP induction depends initially on attaining high levels of Shh signaling, subsequent down-regulation of signaling in presumptive Foxa2^+^ FP cells is required for their differentiation into mature Shh^+^/Foxa2^+^ FP cells; however if this down-regulation is blocked, these cells adopt a p3 fate [5].

Mutant studies in mice illustrate that Shh signaling activity is governed by the balance of Gli activators and repressors [6]. In the absence of Shh, Patched1 (Ptch1) inhibits Smoothened (Smo) to repress signaling through Gli3. Shh binding to Ptch1 alleviates Smo inhibition and initiates signaling to promote Gli-dependent transcription. Gli2 is the main transcriptional activator, whereas Gli1 potentiates Shh signaling as a secondary activator. Proteolytic processing converts Gli3 into the major repressor of Shh signaling, though its full length form acts as an activator. In *Shh* ^−/−^ or *Smo* ^−/−^ neural tubes, FP, p3 and pMN fates are not specified due to the absence of Shh signaling [7], [8]. Elimination of Gli3 repressor function rescues the pMN, but not p3 or FP, fate in these mutants, indicating that the induction of p3 and FP fates depends on Gli activators rather than inactivation of Gli repressors [8], [9]. Consistent with this notion, p3 and FP induction are largely compromised in *Gli2* ^−/−^ and *Gli1* ^−/−^;*Gli2* ^+/−^ embryos [10], [11]. Conversely, ectopic FP and p3 cells are induced in the neural tube of *Ptch1* ^−/−^ embryos with elevated levels of Gli activators [12], [13]. These studies together with the in vivo analysis of intracellular Gli activity [3], [5], [14], [15] clearly unveil the importance of both Gli activators and repressors in the interpretation of the Shh gradient during ventral neural tube patterning. However, the mechanisms by which Shh signaling is converted into graded intracellular Gli activity are poorly understood.

**Figure 1:**
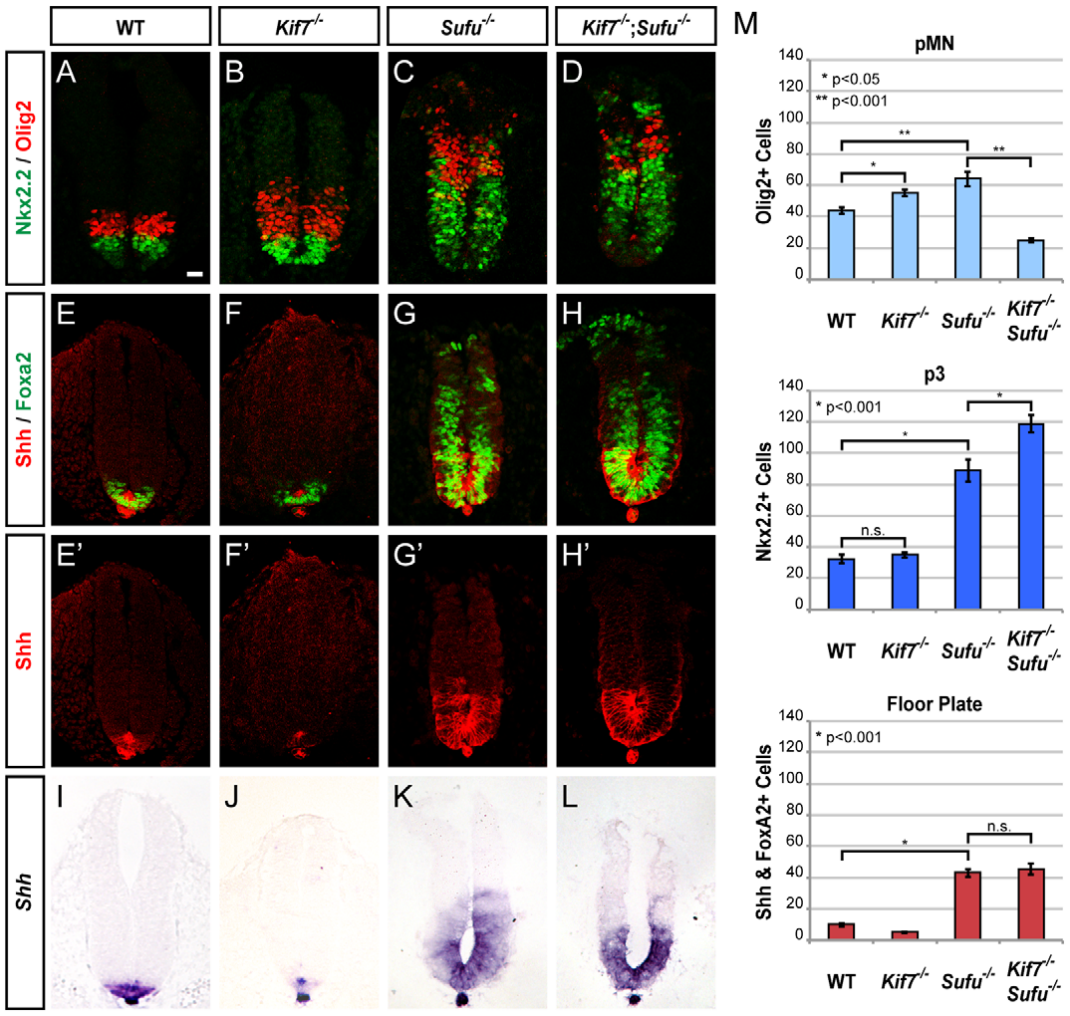
Opposing roles of Kif7 and Sufu in FP development. (**A–D**) Nkx2.2 and Olig2 immunofluorescence labeling shows increasing neural tube ventralization in E9.5 *Kif7* ^−/−^, *Sufu* ^−/−^ and *Kif7* ^−/−^;*Sufu* ^−/−^ embryos. (**E–H**') Shh and Foxa2 expression contrasts the FP deficiencies *Kif7* ^−/−^ embryos to ectopic FP induction in *Sufu* ^−/−^ and *Kif7* ^−/−^;*Sufu* ^−/−^ embryos. *Kif7* ^−/−^;*Sufu* ^−/−^ and *Sufu* ^−/−^ embryos exhibit similar FP defects, indicating that *Kif7* is epistatic to *Sufu* in FP induction. Scale bar, 25 µm. (**I–L**) In situ hybridization analysis of *Shh* RNA expression verifies FP phenotypes. (**M**) Graphs indicate the number of Olig2^+^ pMN, Nkx2.2^+^ p3 and Shh^+^/Foxa2^+^ FP cells, represented as the mean ± SEM, n≥4, n.s., not significant.

Sufu and Kif7 are two key conserved regulators of Gli proteins [16]–[20]. They interact directly with Gli and control their processing, stabilization, as well as subcellular distribution [21]–[25]. *Sufu* ^−/−^ embryos exhibit a severely ventralized neural tube, whereas *Kif7* ^−/−^ embryos display a subtle phenotype with a slight expansion of the pMN domain. It is unknown whether Sufu and Kif7 function together during ventral patterning. Here, we show that they possess cooperative as well as antagonistic functions in the specification of graded Gli activity in the ventral neural tube. Strikingly, when Sufu function is eliminated, diverse ventral cell fates can be specified despite the absence of Shh signaling.

**Figure 2:**
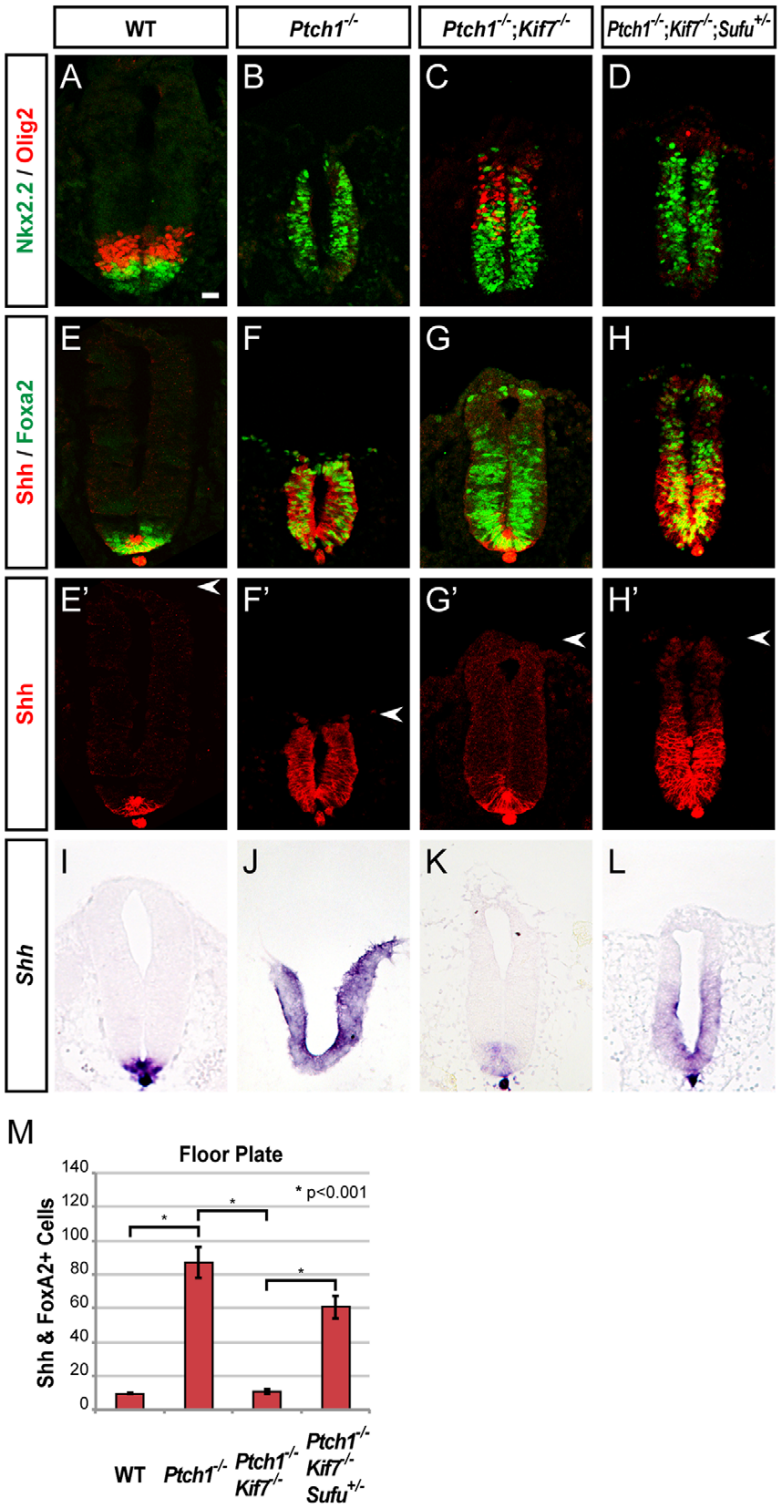
Kif7 promotes ectopic FP induction by restricting Sufu in *Ptch1* mutants. (**A–D**) Nkx2.2 and Olig2 expression shows an increased number of Olig2^+^ pMN cells by *Kif7* inactivation in the *Ptch1* ^−/−^ background. (**E–H**') Shh and Foxa2 expression shows that *Kif7* inactivation abolishes ectopic FP induction in *Ptch1* ^−/−^ embryos, which is restored by reducing one gene dosage of *Sufu*. Arrowheads indicate the dorsal limit of the neural tube. Scale bar, 25 µm. (**I–L**) In situ hybridization analysis of *Shh* RNA expression from the FP in anterior spinal cord sections. (**M**) Graphs indicate the number of FP cells, represented as the mean ± SEM, n≥5.

## Results

### Sufu and Kif7 possess overlapping negative regulatory roles in Shh-dependent ventral neural patterning

To investigate whether Kif7 and Sufu function together during ventral neural patterning, we generated *Kif7* ^−/−^;*Sufu* ^−/−^ mice and examined the expression of Olig2 and Nkx2.2, which are markers of pMN and p3 cells respectively, at embryonic day (E) 9.5. While *Sufu* ^−/−^ embryos exhibit an increase of both Olig2^+^ cells (45% increase) and Nkx2.2^+^ cells (3-fold increase), *Kif7* ^−/−^embryos only show a slight increase (25%) of Olig2^+^ cells (Figures 1A–1C and 1M). These results are consistent with previous observations that both Sufu and Kif7 act as negative regulators of Shh-dependent ventral neural patterning. The fact that *Kif7* inactivation only results in a slight increase of Olig2^+^ cells, but not Nkx2.2^+^ cells, suggests that Kif7 is a weaker negative regulator than Sufu. In *Kif7* ^−/−^;*Sufu* ^−/−^ embryos, we found a significant increase in the number of Nkx2.2^+^ cells (33% higher than that of *Sufu* ^−/−^ embryos) at the expense of Olig2^+^ cells (Figures 1D and 1M), suggesting that the removal of *Kif7* in the *Sufu* ^−/−^ background drives more Olig2^+^ pMN precursors toward the Nkx2.2^+^ p3 fate. These results demonstrate that Sufu and Kif7 possess overlapping roles in the negative regulation of Shh signaling during ventral neural patterning.

**Figure 3:**
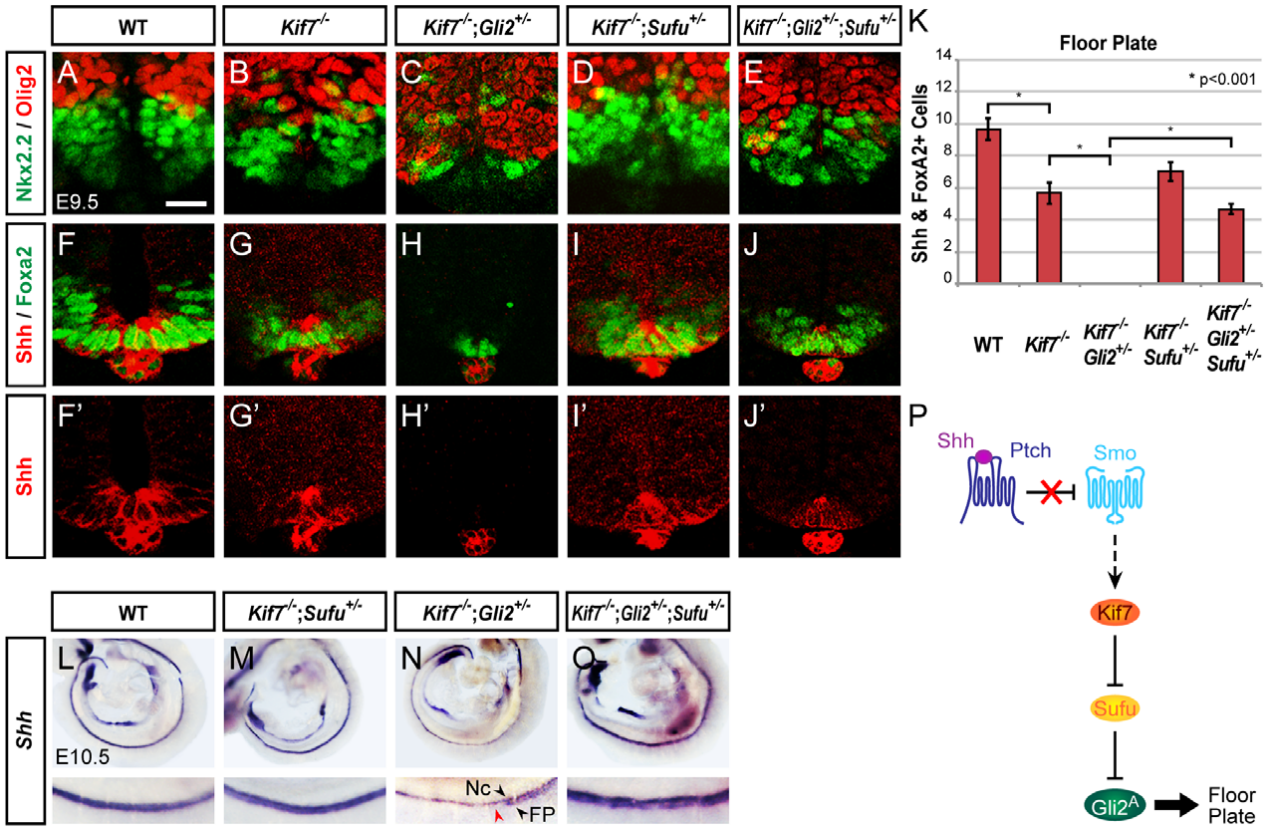
Kif7 alleviates Sufu inhibition of Gli2-dependent FP development. (**A–E**) Nkx2.2 and Olig2 expression shows that *Kif7* inactivation in *Gli2* ^+/−^ mice results in a ventral shift of the Olig2^+^ pMN and Nkx2.2^+^ p3 domains, and reduced formation of p3 cells. The dorsalization and reduced p3 cell specification of *Kif7* ^−/−^;*Gli2* ^+/−^ embryos is restored to that of *Kif7* ^−/−^ embryos by reducing *Sufu* gene dosage. (**F–J**') Shh and Foxa2 expression shows *Kif7* inactivation in *Gli2* ^+/−^ embryos leads to FP defects, which is rescued in *Kif7* ^−/−^;*Gli2* ^+/−^;*Sufu* ^+/−^ embryos by the reduction in *Sufu* gene dosage. Scale bar, 25 µm. (**K**) Graphs indicate the number of FP cells, represented as the mean ± SEM, n = 3. (**L–O**) Whole mount in situ hybridization of *Shh* RNA expression from the notochord (Nc) and FP showing that FP defects in *Kif7* ^−/−^;*Gli2* ^+/−^ embryos are rescued by the reduced *Sufu* gene dosage of *Kif7* ^−/−^;*Gli2* ^+/−^;*Sufu* ^+/−^ embryos. (**P**) Model of Kif7 and Sufu regulation of Gli2 during FP development: Kif7 restricts the potent inhibitory action of Sufu on Gli2 to promote FP induction.

### Opposing roles of Kif7 and Sufu in FP development

Kif7 appears to act positively in Shh-dependent FP induction [17], [18]. The number of Shh^+^/Foxa2^+^ FP cells is reduced by 50% in E9.5 *Kif7* ^−/−^ embryos (Figures 1E–F and 1 M). In contrast, *Sufu* ^−/−^ embryos exhibit a drastic increase (3-fold) in the number of FP cells (Figures 1G–1G' and 1M), revealing a key role for Sufu in limiting Shh-dependent FP induction. These results illustrate that Kif7 and Sufu play opposing roles in FP development. Importantly, *Kif7* ^−/−^;*Sufu* ^−/−^ embryos show a FP defect similar to that of *Sufu* ^−/−^ embryos (Figures 1G–H' and 1M). In situ hybridization analysis confirms that the expansion of the Shh domain in these mutants is due to increased *Shh* RNA expression (Figures 1I–1L). Although Kif7 is positively involved in FP induction, it exerts no effect on ectopic FP development in *Sufu* ^−/−^ embryos as revealed by the analysis of *Kif7* ^−/−^;*Sufu* ^−/−^ embryos. These observations indicate that *Kif7* is epistatic to *Sufu* in FP induction and suggest that the positive action of Kif7 in FP induction is mediated through the restriction of Sufu.

**Figure 4:**
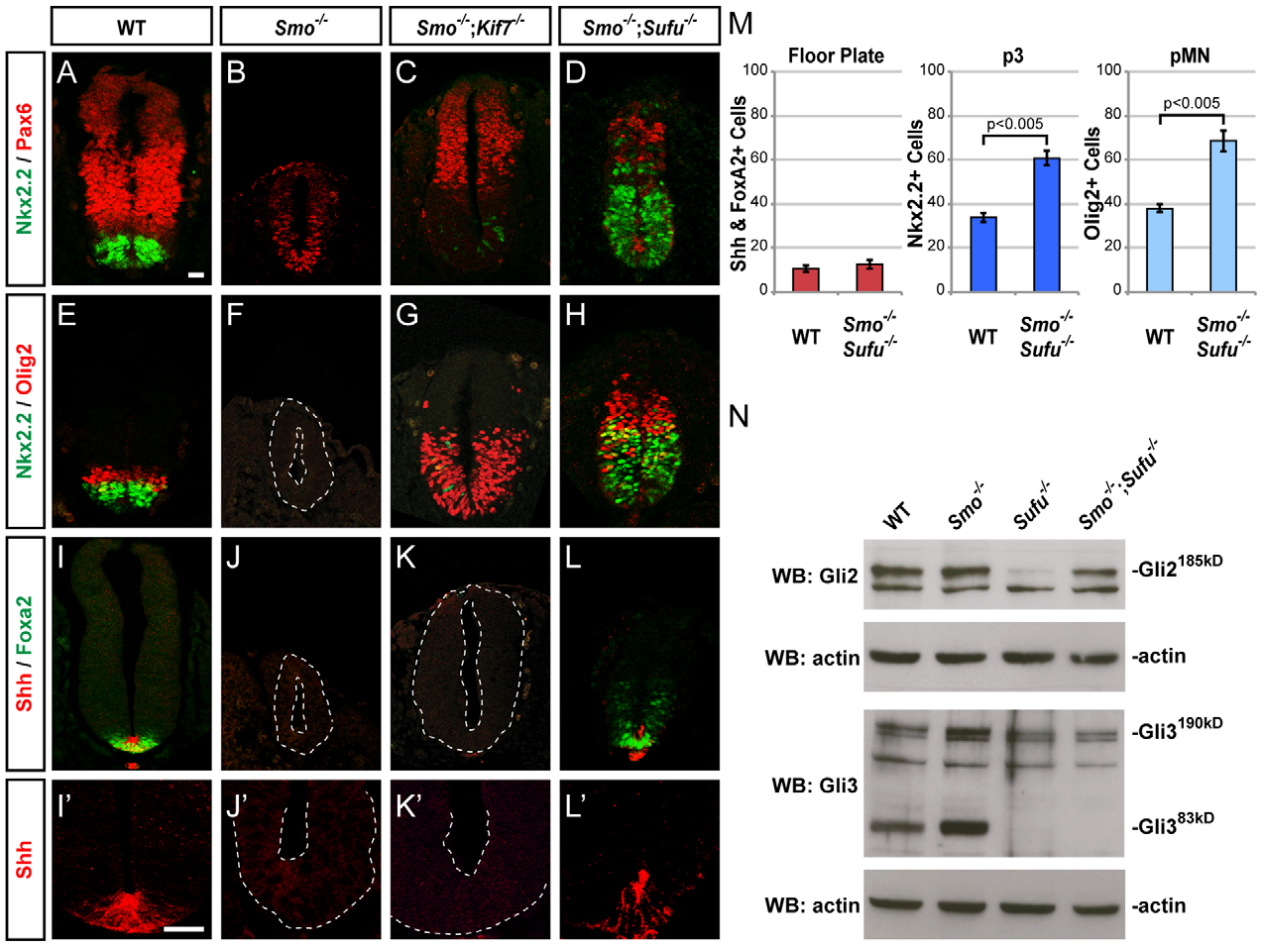
Diverse ventral fates form despite the absence of graded Shh signaling when Sufu function is abolished. (**A–D**) Nkx2.2 and Pax6 expression shows that *Kif7* or *Sufu* inactivation rescues dorsalization of the *Smo* ^−/−^ neural tube. (**E–H**) Nkx2.2 and Olig2 expression shows that in *Smo* ^−/−^ embryos, *Kif7* inactivation rescues Olig2^+^ pMN cell specification, but rarely that of Nkx2.2^+^ p3 cells, while *Sufu* inactivation leads to robust induction of both cell fates. (**I–L**') Shh and Foxa2 expression shows that only *Sufu* inactivation, but not that of *Kif7*, rescues FP development in *Smo* ^−/−^ embryos. Scale bar, 25 µm. (**M**) Graphs indicate the number of pMN, p3 and FP cells, represented as the mean ± SEM, n≥4. (**N**) Western blot analysis shows reduced Gli2 expression in *Sufu* ^−/−^ embryos are restored when *Smo* is simultaneously inactivated; little to no Gli3^83kD^ repressor is expressed in *Sufu* ^−/−^ and *Smo* ^−/−^;*Sufu* ^−/−^ embryos.

### *Kif7* is required for ectopic FP induction in *Ptch1* mutants

Next, we examined how Kif7 acts positively in ectopic FP induction in *Ptch1* ^−/−^ mice, where Smo is constitutively active. Previously, we have shown that Shh^+^ FP cells are formed throughout the neural tube of E9.5 *Ptch1* ^−/−^ embryos [13] (see Figures 2A–2B and 2E–2F'). Gli2 is the major activator of mammalian Shh signaling and is essential for FP development [10]. Consistent with this notion, removal of Gli2 function largely suppresses ectopic FP development in the *Ptch1* ^−/−^ neural tube [13]. If Kif7 acts as a positive regulator of Shh signaling, we expect that *Ptch1* ^−/−^;*Kif7* ^−/−^ embryos should exhibit a FP phenotype similar to that of *Ptch1* ^−/−^;*Gli2* ^−/−^ embryos. Indeed, removal of Kif7 function drastically reduces the number of Shh^+^/Foxa2^+^ FP cells by 90% in the *Ptch1* ^−/−^ background (Figures 2G–2G' and 2M), suggesting that Kif7 is required to maintain robust Gli2 activator function for FP induction.

**Figure 5:**
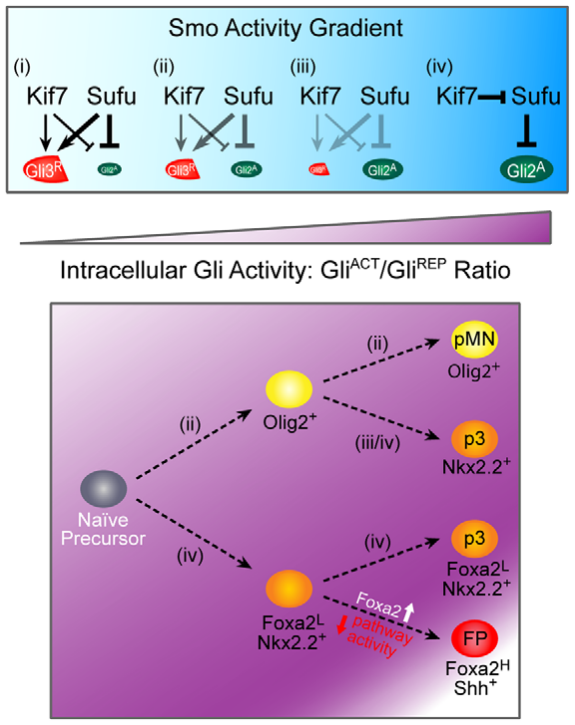
A model of Kif7 and Sufu regulation of graded Gli activity in ventral cell fate specification. The concerted actions of Kif7 and Sufu alter the Gli activator to repressor ratio to regulate intracellular Gli activity for the specification of ventral cell fates (pMN, p3 and FP) from naïve neuroepithelial precursor cells. As Shh signaling increases Smo activity, Gli repressor formation and the inhibition of activators become less efficient as denoted (**i–iii**), while robust Smo activation results in Kif7 antagonizing the inhibition of Gli activators by Sufu for p3 and FP induction (**iv**).

Our analysis indicates that Kif7 acts upstream of Sufu during FP induction (Figure 1). To test whether activated Smo regulates Sufu through Kif7, we examined the effects of reduced *Sufu* gene dosage on FP induction in *Ptch1* ^−/−^;*Kif7* ^−/−^ embryos. Consistent with this, *Ptch1* ^−/−^;*Kif7* ^−/−^;*Sufu* ^+/−^ embryos show a 6-fold increase in the number of FP cells when compared with *Ptch1* ^−/−^;*Kif7* ^−/−^ embryos (Figures 2H–H', 2I–L, 2M). These observations support the model that Sufu is the major negative regulator of FP induction and that activated Smo restricts the inhibitory function of Sufu through Kif7.

Although Kif7 appears to act negatively in the induction of neuronal progenitors (Figure 1), a positive regulatory role for Kif7 is also unveiled in ventral neural patterning of *Ptch1* ^−/−^;*Kif7* ^−/−^ and *Ptch1* ^−/−^;*Kif7* ^−/−^;*Sufu* ^+/−^ embryos (Figure 2). When compared with *Ptch1* ^−/−^ embryos, *Ptch1* ^−/−^;*Kif7* ^−/−^ embryos exhibit a dramatic increase in the number of Olig2^+^ cells and fewer Nkx2.2^+^ cells (Figures 2B–2C), suggesting that activated Smo is less efficient at driving cells towards the Nkx2.2^+^ p3 fate in the absence of Kif7. The fact that *Ptch1* ^−/−^;*Kif7* ^−/−^;*Sufu^+/^* ^−^ embryos show a reduction in the number of Olig2^+^ pMN cells further suggests the involvement of Sufu in limiting the conversion of pMN to p3 fate in Smo-active cells. Together, these observations indicate that when Smo is active, Kif7 functions positively in the induction of both non-neuronal FP and neuronal p3 cells.

### Kif7 alleviates Sufu inhibition of Gli2-dependent FP development

Gli2 is essential for robust Shh signaling and FP induction [10]. We have previously demonstrated that reduction of one dose of *Gli2* can exacerbate the FP phenotype of *Kif7* ^−/−^ mice [17] (see Figures 3A–C, 3F–H', 3L–N and 3K). To determine whether the absence of FP cells in *Kif7* ^−/−^;*Gli2* ^+/−^ embryos is due to elevated inhibition by Sufu, we reduced the gene dosage of *Sufu* to test the possibility of restoring FP development. Indeed, FP cells are readily detected along the neural tube of *Kif7* ^−/−^;*Gli2* ^+/−^;*Sufu^+/^* ^−^ embryos (Figures 3J–J', 3O and 3K). Furthermore, when the *Sufu* dosage is reduced in *Kif7* ^−/−^;*Gli2* ^+/−^ embryos, the number of Nkx2.2^+^ p3 cells is restored to a level comparable to that found in *Kif7* ^−/−^ embryos (Figures 3B–3E). These results indicate that the requirement for Kif7 in promoting robust Gli2 activity can be bypassed by reducing the amount of Sufu and suggest that the primary function of Kif7 is to relieve the inhibitory effect of Sufu on Gli2 (Figure 3P).

### Diverse ventral cell fates are specified despite the absence of graded Shh signaling when Sufu function is eliminated

Our results so far indicate that when Smo is activated, Kif7 regulation of Sufu's inhibitory action on Gli2 is a critical step in Shh signaling. We next investigated the roles of Kif7 and Sufu in cells lacking Smo, in which the pathway is repressed and insensitive to Hh inputs. We have previously shown that *Kif7* inactivation in the *Smo* ^−/−^ background alleviates pathway inhibition, leading to the induction of Olig2^+^ pMN cells in *Smo* ^−/−^;*Kif7* ^−/−^ mice [17] (see Figures 4F and 4G). However, despite the abundance of Gli2 proteins in *Smo* ^−/−^;*Kif7* ^−/−^ embryos [17], Gli2 rarely specifies Nkx2.2^+^ p3 cells and is incapable of inducing FP without activated Smo (Figures 4G and 4K–K'). Strikingly, pMN, p3 and FP cells are all detected in *Smo* ^−/−^;*Sufu* ^−/−^ embryos (Figures 4D, 4H and 4L–L'). Though the number of pMN and p3 cells is higher than in wild type embryos (Figure 4M), the relative position of these neuronal progenitor and FP cells along the dorsoventral axis of the neural tube appears quite normal. Consistent with the observations that Sufu controls Gli2 degradation and Gli3 processing [22], [23], [25], we found that *Sufu* ^−/−^ embryos exhibit low levels of Gli2^185kD^ and little or no Gli3^83kD^ (Figure 4N). Importantly, Gli2^185kD^ is restored to higher levels in *Smo* ^−/−^;*Sufu* ^−/−^ embryos. These observations indicate that, in the absence of Sufu, diverse cell fates (pMN, p3 and FP) can be specified despite the absence of graded Shh signaling. Thus, a Shh signaling gradient is no longer necessary to drive graded intracellular Gli activity responsible for the specification of distinct neuronal and non-neuronal cell fates when the inhibitory action of Sufu is eliminated.

## Discussion

In this study, we establish that Kif7 and Sufu play distinct and overlapping functions in specifying graded Gli activity during Shh-dependent ventral neural tube patterning. We show that there are different requirements of Kif7 and Sufu for FP, p3 and pMN cell fate specification. Kif7 acts positively in Shh-dependent FP induction and negatively in the formation of pMN cells, while its dual functions are involved in p3 fate specification. In contrast, Sufu functions universally as a negative regulator of these Shh-induced cell fates. Importantly, in the complete absence of extracellular Shh signaling (i.e. in *Smo* ^−/−^ embryos), inactivation of *Kif7* or *Sufu* can lead to specification of distinct ventral cell fates. We propose that, through acting on both Gli activators and repressors, Sufu and Kif7 function cooperatively to generate graded intracellular Gli activity (Figure 5).

Sufu possesses multiple molecular functions in the negative regulation of Shh signaling. It limits the nuclear translocation of Gli activators by forming inhibitory complexes in the cytoplasm [21], [26], [27] and also plays a major role in the processing of Gli3 into its repressor form [22], [23]. Studies using cultured fibroblasts show that Smo activation promotes the dissociation of inhibitory cytoplasmic Sufu-Gli complexes [24], [25]. The molecular action of Kif7, a member of the kinesin motor proteins, is not well understood. The motor domain of Kif7 is important for its Shh-dependent translocation to the primary cilium [19]. When Smo is inactive or absent, Kif7 is critical for Gli3 repressor function as loss of Kif7 restores the formation of pMN cells in *Smo* ^−/−^ embryos similar to those observed in *Gli3* ^−/−^;*Smo* ^−/−^ embryos [8], [17]. Our recent studies suggest that Kif7 restricts the ciliary localization of Sufu-Gli complexes in chondrocytes [28]. Here, we provide genetic evidence that Kif7 acts positively in Shh-dependent FP induction by alleviating the inhibitory action of Sufu on Gli2. We speculate that Kif7 mediates the action of activated Smo to promote the dissociation of inhibitory Sufu-Gli complexes. Contrary to this Sufu-dependent positive regulatory function, Kif7 acts negatively in Shh signaling via both Sufu-dependent and -independent mechanisms. We show here that more neuronal precursors adopt a p3 fate in *Kif7* ^−/−^;*Sufu* ^−/−^ embryos than those in *Sufu* ^−/−^ embryos, indicating that loss of Kif7 augments Hh pathway activity in a *Sufu* ^−/−^ background. Further studies are needed to decipher these Sufu-dependent and -independent functions of Kif7 in the control of Gli activator and repressor.

Strikingly, inactivation of *Sufu* leads to the specification of diverse ventral cell fates in the absence of extracellular Shh signaling. In *Smo* ^−/−^;*Sufu* ^−/−^ embryos, not only pMN, but p3 and FP fates are specified with relatively normal positions along the dorsoventral axis of the neural tube. Thus, positional information and distinct cell fates in the ventral neural tube can be conveyed without Smo-dependent signaling when Sufu is absent. These observations suggest that Sufu is the key regulatory target of Shh signaling and that its regulated activity leads to distinct intracellular Gli activity and cell fate. In the absence of Sufu, Gli3 repressor is not formed and Gli activators are not restricted by cytoplasmic sequestration. In this situation, Smo activation is no longer required to promote Gli2-dependent formation of FP and p3 cells. In addition to Shh, retinoic acid and Tcf signaling also contribute to the patterning of the ventral neural tube [29]–[33]. It remains to be determined how these and other signaling pathways contribute to the regulation of Gli activators. Perhaps once Sufu regulation is alleviated, alternate pathways normally obscured by Shh can assume more prevalent roles in patterning, and through the cross-regulatory interactions of the *Nkx2*.2, *Olig2* and *Pax6* gene networks, can refine the precise patterning of these ventral progenitor domains [15].

## Materials and Methods

### Ethics Statement

All experimental procedures performed were approved by The Hospital for Sick Children Animal Care Committee.

### Mice

*Sufu* mutant mice were generated by crossing NLS-Cre mice with ubiquitous Cre expression (provided by C. Lobe, University of Toronto) to *Sufu*-floxed mice [34]. *Kif7* [17], *Smo* [35], *Gli2* [36] and *Ptch1* [12] mutant mice were maintained in a CD-1 outbred background and genotyped as described.

### Immunofluorescence

Embryos were harvested at E 9.5 and fixed in 4% paraformaldehyde (PFA) in PBS at 4°C overnight, then subjected to ethanol series dehydration, paraffin embedding and sectioning at 7 µm. Immunohistochemistry on anterior spinal cord sections was performed as described [13]. Mouse anti-Nkx2.2 and anti-Foxa2 (HNF-3ß) (1∶20, Developmental Studies Hybridoma Bank), rabbit anti-Olig2 (1∶300, Chemicon), rabbit anti-Pax6 (1∶300, Covance), and rabbit anti-Shh (1∶50, Santa Cruz) antibodies were used. Images were acquired with a Zeiss LSM510 META laser scanning confocal microscope.

### Statistical Analysis

All analysis was performed using anterior spinal cord sections at the forelimb bud level in somite-matched E9.5 embryos (24±1 somite pairs), except *Ptch1* single mutant embryos which arrest at 16–20 somite pairs. Cell fate quantification data were expressed and plotted as means ± standard error of the mean (SEM). Statistical analysis for multiple comparisons was completed using one-way ANOVA followed by the Bonferroni post-test (Figures 1M, 2M and 3K). Analysis for pair-wise comparisons was completed using the Student's *t*-Test (Figure 4M). The sample size and p-value for each Bonferroni comparison or Student's *t*-Test are provided in the respective legends and figures.

### In situ hybridization

Whole-mount or section in situ hybridization was performed as described [10]. Embryos were fixed in 4% PFA in PBS at 4°C overnight, then subjected to methanol series dehydration. In situ hybridization was carried out with digoxigenin-dUTP-labeled RNA probes for *Shh* [36].

### Western blot analysis

Embryos were snap-frozen, and sonicated in RIPA buffer as described [17]. Immunoblotting was performed with rabbit anti-Gli3 (Santa Cruz), and rabbit anti-Gli2 (amino acids 327–442) generated using standard protocols.
